# Transcription profile of a human breast cancer cell line expressing MMTV-like sequences

**DOI:** 10.1186/1750-9378-1-7

**Published:** 2006-12-15

**Authors:** Mariana Fernandez-Cobo, Stella M Melana, James F Holland, Beatriz GT Pogo

**Affiliations:** 1 Department of Medicine, Mount Sinai School of Medicine, New York, USA; 2 Department of Microbiology, Mount Sinai School of Medicine, New York, USA

## Abstract

**Background:**

It has been postulated that inflammation caused by certain viruses might result in cancer. Recently, it was shown that childhood lymphoblastic leukemia, breast and ovarian cancers express an interferon-related signature, providing support for this notion. We have previously shown that 38% of the sporadic breast cancers contain MMTV-like *env *gene sequences. To find out if the presence and expression of MMTV-like sequences correlated with an inflammatory phenotype, we have compared the expression profile of two sublines of MCF-7 cells, one containing the MMTV-like sequences (*env*+), the other one lacking them (*env*-).

**Results:**

The results indicated that there were 47 differentially expressed genes between the two sublines. Among 27 upregulated genes in the *env*+ cells there were 7 interferon-related genes, 5 TNF-connected genes and 2 TGFβ-related genes.

**Conclusion:**

These results suggest that the *env*+ cells were most likely responding to an infectious agent, and support the hypothesis that a viral infection may play a role in breast cancer pathogenesis.

## Background

We and others [1-4] have shown that 37 to 41% of sporadic breast cancer samples contain MMTV-like *env *gene sequences. The sequences are expressed as RNA [5] and as protein in breast cancers (Melana *et al*., submitted). They are absent from the normal breasts of patients with *env *positive tumors [6] and are expressed as RNA exclusively in the cancer cells [7]. The whole proviral structure, designated human mammary tumor virus (HMTV), which has 95% homology to MMTV, can be detected in two tumors [8]. Although sequence variations are observed in the C-terminal of human *sag *sequences, the cloned human *sag *sequences expressed in human B lymphocytes can activate human T-cells, as can the mouse Sag, indicating that it can be functional [9]. Moreover, viral particles with the morphological characteristics of betaretroviruses are observed in primary cultures of human beast cancer [10]. Taken together, these results suggest that an infectious agent is present in some human breast cancers.

Chronic inflammation has been implicated in tumor progression. New evidence suggests that the inflammation caused by certain viruses results in cancer [Reviewed in [11]]. Recently, it was reported that childhood lymphoblastic leukemia, as well as breast and ovarian cancers express an interferon-related signature, but not found in other human cancers studied [12]. This finding provides molecular support for the role of inflammation or viral infection in cancer pathogenesis [12].

The established breast cancer cell line MCF-7 is widely used in research, and many subclones are available. Some of the original isolates produce retroviral-like particles [13]. Furthermore, May and Westerly [14] described the presence of an MMTV-like 6.6 Kb EcoR1 fragment in some of the MCF-7 cell lines, which was absent in other breast cancer lines and in normal tissue.

Continuous passage with subsequent chromosomal change [15] may have eliminated viral sequences from some of them. It has been reported that some sublines of MCF-7 show biological differences [16] and significant genetic variation in RNA expression [17,18].

We have previously reported that a subline of MCF-7 containing *env *and LTR sequences [19,20] and that it expressed the *env *gene as RNA [7], while other sublines were negative for *env *gene [[21] and our own results]. To find out whether the presence of viral sequences is related to an interferon-related signature, we have compared the expression profiles of two sublines of MCF-7 [22], one which contains the MMTV-like *env *gene sequence (*env*+) and one which lacks it (*env*-).

## Results

**Figure 1:**
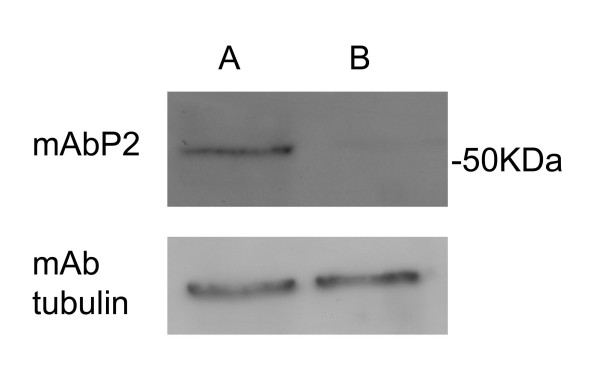
Western blot of MCF-7 cells. Experimental conditions as described in Materials and Methods. A: MCF-7 (+) cells; B: MCF-7(-) cells.

The presence of the Env protein was investigated in both sublines. In Fig. 1 the result of the immunoblotting experiment is shown. The HMTV *env+ *cell line expressed a protein of a MW of approximately 50 kD which reacted with mAbP2, a monoclonal antibody against a synthetic peptide derived from human *env *sequences (Melana *et al *submitted). It was absent in the HMTV *env*- cells. Tubulin was equally present in both extracts.

The results of the cDNA arrays are shown in Tables 1 and 2. Nineteen genes showed a > 2.5 fold difference in their adjusted intensity between HMTV *env*+ and *env*- cells, while another eight genes were only expressed in the HMTV *env*+ cells (Table 1). Twenty genes were downregulated (Table 2) in HMTV *env *+ cells. Taken together, there were 47 differentially expressed genes. Among the 27 upregulated genes there were six interferon-inducible ones: IFI6, TRIM22, IFITM1, IFITM2+IFITM3, IFI27 and IP-30 and a receptor IFNGR2. In addition, there were five upregulated genes that have a connection with TNF or are involved in its signaling, like LTBR, TRAF3, MMP17, PKN1 and MAPK13. The cytokine TGFβ, and its downstream effector early growth response protein 1 (EGR1), were also upregulated in *env*+ cells. Twenty genes were down regulated in HMTV *env+ *cells.

**Table 1:** Up-regulated genes in HMTV *env*+ cells

**Acc #**	**Gene symbol**	**Protein/gene**	**Ratio**	**Diff**
X02492	IFI6	Interferon-inducible protein 6	12.44	4108
J05633	ITGB5	Integrin beta 5	9.46	3401
X82200	TRIM22	Tripartite motif-containing 22	5.76	1495
L03840	FGFR4	Fibroblast growth factor receptor 4	5.72	1742
J04164	IFITM1	Interferon induced transmembrane protein 1 (9–27)	5.38	6776
M64595		small G protein	4.74	1720
X57351	IFITM2 + IFITM3	interferon induced transmembrane prot 2 (1–8D) + 3 (1–8U)	4.25	1082
X89576	MMP17	matrix metalloproteinase 17	4.20	1647
X52541	EGR1	early growth response protein 1	3.39	1327
L29220	CLK3	CDC-like kinase 3	3.38	1521
X66362	PCTK3	PCTAIRE protein kinase 3	3.34	1368
U33053	PKN1	protein kinase N1	2.85	1075
L04270	LTBR	lymphotoxin beta receptor (TNFR superfamily, member3)	2.79	1406
U09579	CDKN1A	cyclin-dependent kinase inhibitor 1A (p21, Cip1)	2.69	6193
U14966	RPL5	60S ribosomal protein L5	2.68	1641
M29039	JUNB	jun-B	2.65	1313
M29971	MGMT	6-O-methylguanine-DNA methyltransferase	2.63	1971
M65199	EDN2	endothelin 2	2.58	1535
U57342	MLF2	myeloid leukemia factor 2	2.52	2897
X69398	CD47	CD47 glycoprotein; integrin-associated protein	Up	4152
U12255	FCGRT	Fc fragment of IgG, receptor, transporter	Up	2960
AF004709	MAPK13	mitogen-activated protein kinase 13	Up	2049
X02812	TGFB	transforming growth factor, beta 1	Up	1535
U21092	TRAF3	TNF receptor-associated factor 3	Up	1407
U05875	IFNGR2	interferon gamma receptor 2	Up	1372
X67325	IFI27	interferon, alpha-inducible protein 27	Up	1182
J03909		gamma-interferon-inducible protein; IP-30	Up	1076

**Table 2:** Down-regulated genes in HMTV *env*+ cells

**Acc #**	**Gene symbol**	**Protein/gene**	**Ratio**	**Diff**
U02687	FLT3	fms-related tyrosine kinase 3	-6.20	-2143
X74295	IGA7B	integrin alpha 7B	-5.13	-2249
X53587	ITGB4	integrin beta 4	-4.80	-2384
M34671	CD59	CD59 molecule, complement regulatory protein	-3.60	-2551
L25081	RHOC	ras homolog gene family, member C	-3.59	-2341
U89278	PHC2	polyhomeotic-like 2	-3.53	-1250
X16277	ODC1	ornithine decarboxylase 1	-2.87	-1295
AF029670	RAD51C	RAD51 homolog C	-2.61	-1713
M20430	HLA-DRB1	MHC class II HLA-DR-beta	Down	-4288
J04111	JUN	c-jun proto-oncogene; transcription factor AP-1	Down	-1745
U70310	FANCG	DNA repair protein XRCC9	Down	-1514
M59911	ITGA3	integrin alpha 3	Down	-1393
M97934	STAT2	signal transducer and activator of transcription 2	Down	-1248
L38518	SHH	sonic hedgehog	Down	-1183
X51521	VIL2	ezrin; villin 2	Down	-1177
L07515	CBX5	chromobox homolog 5; heterochromatin protein homolog 1 (HP1)	Down	-1153
M15400	RB1	retinoblastoma 1	Down	-1109
M54995	PPBP	pro-platelet basic protein	Down	-1100
X54199	GART	trifunctional purine biosynthetic protein adenosine 3	Down	-1092
U47686	STAT5 A +B	signal transducer and activator of transcription 5 A+B	Down	-1072

## Discussion

Comparison of the expression profiles of sublines derived from the same cell line provides an excellent model with minimal differences. Karyogenetic analysis revealed that the two sublines have similar complex chromosomal patterns (not shown). The comparison of expression profiles of MCF-7 *env+ *and *env*- cells indicated preferential expression of interferon-related genes: 26% (7/27) of the up-regulated genes. These differences may indicate a trend. Einav *et al*. [12] have reported that 40% of clinical breast cancer samples display an interferon-associated signature; 17 out of 36 (47%) of the upregulated genes. Our results are consistent with, but cannot be directly compared with those of Einav's for several reasons: we used only one cell line for analysis, the participation of stroma and surrounding tissues has been eliminated from our study, and finally, we used a different set of arrays. Nevertheless, our results strongly indicate that HMTV *env+ *MCF-7 cells express more interferon-related genes than the HMTV *env*- MCF-7 cells, suggesting that they may be responding to an infectious agent as proposed by Einav *et al*. [12]. The expression profile of HMTV *env*+ cells suggests an increased potential for cell growth, a fact that may be related to their more malignant phenotype as has been described in breast cancer cells associated with HMTV [23,3,24]. It is remarkable that the alpha 7 and beta 4 integrins were significantly down regulated in *env*+ cells, as has been reported in a set of finite life-span metastatic breast cancer cells which were also *env*+ [25].

Whether the HMTV works as initiator and/or as promoter of malignant growth is uncertain. Molecular evidence that HMTV expression is responsible for the increase in interferon-related expression is being sought.

## Conclusion

The results clearly indicate that the transcriptional profile of the cells expressing HMTV sequences is enriched in genes involved in inflammation process. This finding is significant because it was obtained comparing cells derived from the same cell line that have similar genetic background and minimal expressing differences. This supports the hypothesis that a viral infection may play a role in breast cancer pathogenesis.

## Methods

MCF-7 cells were obtained from American Type Culture Collection (ATCC) and were propagated in vitro as recommended by the provider and as described in previous publications (1, 5). To determine whether the viral protein was expressed in our MCF-7 cells, western blotting was used. Protein lysates were prepared from approximately 1 × 10^7 ^cells. Equal amounts of protein from each sample were loaded onto an SDS-PAGE-10% polyacrylamide gel, followed by transfer to PVDF membranes. Western blot analysis was performed using mAbP2 (a monoclonal antibody against a peptide of the Env protein), and mAb-tubulin as primary antibodies (Sigma Aldrich). Proteins were visualized using horseradish peroxidase-labeled sheep anti-mouse IgG (GE Healthcare Bio-Sciences Corp.) as a secondary antibody followed by enhanced chemiluminescence (GE Healthcare Bio-Sciences Corp.).

The expression profile was studied using the Atlas Human Cancer 1.2 cDNA expression array; a nylon membrane printed with 200–600 bp long fragments of 1176 characterized genes involved in cancer, 9 housekeeping genes and 6 negative controls (Clontech, CA). These conditions were described in detail in a previous publication [25]. Briefly, RNA was extracted and labeled with Atlas pure total RNA labeling system and hybridized to an Atlas Human Cancer 1.2 cDNA expression array (Clontech, CA) according to the manufacturer's instructions. Both cell sublines were probed twice in separate assays, and the accuracy of each duplicate was assessed by Pearson's correlation coefficient based on the adjusted intensity of all genes spotted on the membrane.

Hybridizations with 30 μg of total RNA were performed according to the manufacturer instructions. The hybridized membranes were exposed onto a phosphorimager screen and were read using a phosphorimager reader (Molecular Dynamics). The scanned images were aligned and analyzed using AtlasImage 2.01 software (Clontech). When averaging or comparing samples, the adjusted intensity signal was normalized using the global normalization mode featured in the software. We reported only those genes whose ratios of differential expression were 2.5-fold or more, or genes that were undefined for one type of sample, but were detected on the other. (Undefined genes are those whose intensity were below the signal threshold) In the later event, when we lack a numerical value for the ratio, it was defined as being "up" or "down". Furthermore, for each gene we stated the difference (diff) in adjusted normalized intensity between the two cell lines.

Accession number (Acc#), gene symbol and protein or gene name are according to GeneBank.
